# The role of immunometabolism in macrophage polarization and its impact on acute lung injury/acute respiratory distress syndrome

**DOI:** 10.3389/fimmu.2023.1117548

**Published:** 2023-03-20

**Authors:** Lian Wang, Dongguang Wang, Tianli Zhang, Yao Ma, Xiang Tong, Hong Fan

**Affiliations:** ^1^ Department of Respiratory and Critical Care Medicine, West China Hospital, Sichuan University, Chengdu, China; ^2^ Department of Geriatrics and National Clinical Research Center for Geriatrics, West China Hospital, Sichuan University, Chengdu, China

**Keywords:** macrophage polarization, polarization regulation, immunometabolism, metabolic reprogramming, acute lung injury, acute respiratory distress syndrome

## Abstract

Lung macrophages constitute the first line of defense against airborne particles and microbes and are key to maintaining pulmonary immune homeostasis. There is increasing evidence suggesting that macrophages also participate in the pathogenesis of acute lung injury (ALI)/acute respiratory distress syndrome (ARDS), including the modulation of inflammatory responses and the repair of damaged lung tissues. The diversity of their functions may be attributed to their polarized states. Classically activated or inflammatory (M1) macrophages and alternatively activated or anti-inflammatory (M2) macrophages are the two main polarized macrophage phenotypes. The precise regulatory mechanism of macrophage polarization is a complex process that is not completely understood. A growing body of literature on immunometabolism has demonstrated the essential role of immunometabolism and its metabolic intermediates in macrophage polarization. In this review, we summarize macrophage polarization phenotypes, the role of immunometabolism, and its metabolic intermediates in macrophage polarization and ALI/ARDS, which may represent a new target and therapeutic direction.

## 1 Introduction

Acute lung injury (ALI) is a common and critical disease caused by a variety of direct or indirect factors, including severe infection, pancreatitis, shock, trauma, major surgery, and ischemia–reperfusion (1–3). ALI usually leads to acute respiratory distress syndrome (ARDS), a more severe clinical manifestation, and the terminal pathophysiological characteristics are remarkably similar. ALI/ARDS is characterized by excessive and uncontrolled inflammatory responses to lung injury, leading to generalized epithelial and endothelial barrier injury, alveolar-capillary membrane dysfunction, increased vascular permeability, alveolar hemorrhage, and diffuse alveolar damage (3–7). The clinical manifestations include severe hypoxemia, diffuse bilateral pulmonary infiltration, and pulmonary edema (8). ALI is associated with high morbidity and poor prognosis, with an age-adjusted incidence of 86.2/100,000, a mortality rate of 38.5%, and persistent pulmonary dysfunction in 50% of survivors (9, 10). Currently, there is no effective therapy to reduce mortality or improve the prognosis of patients with ALI/ARDS. Hence, researching the pathogenesis and identifying the signaling pathways of ALI/ARDS could help provide novel targets for therapeutic intervention.

Macrophages play a significant role in innate immunity by serving as heterologous phagocytes and by expressing pattern recognition receptors (11). Lung macrophages constitute the first line of defense against airborne particles and microbes and are key to maintaining pulmonary immune homeostasis (11). There is increasing evidence suggesting that macrophages also participate in the pathogenesis of ALI/ARDS, including the modulation of inflammatory responses and the repair of damaged lung tissues (12, 13). During the development of the inflammatory response, macrophages exert a pro-inflammatory effect in the early stages and play an anti-inflammatory role in the later stages. The diversity of their functions may be attributed to their polarized phenotypes; however, the precise regulatory mechanisms of macrophage polarization remain incompletely understood and involve a range of signaling pathways and transcriptional and post-transcriptional regulatory networks (13–16). Phenotypic and functional changes in macrophages are accompanied by dramatic shifts in cell metabolism, an emerging research field termed immunometabolism, also known as metabolic reprogramming. Pro-inflammatory polarization of macrophages is associated with increased glycolysis and a shift toward the pentose phosphate pathway (PPP) and fatty acid synthesis (17–19). However, anti-inflammatory macrophages primarily use oxidative phosphorylation (OXPHOS), glutamine metabolism, and fatty acid oxidation (FAO) (19, 20). Moreover, pro- and anti-inflammatory macrophages are characterized by specific pathways that regulate lipid and amino acid metabolism and affect their responses (18). These metabolic adaptations are necessary to support macrophage activity and maintain polarization in specific contexts. The regulation of immune metabolism to affect macrophage polarization may be a novel direction for the treatment of ALI. In this review, we summarize macrophage polarization phenotypes, the role of immunometabolism in macrophage polarization, and its impact on ALI/ARDS.

## 2 Macrophage subsets

Macrophages are a major group of innate immune cells, which exist in various tissues with significant heterogeneity and phenotypic specialization (21). They are regulated in a tissue-specific manner and play a significant role in phagocytosis and digestion of pathogens and infected and apoptotic cells and can recruit and regulate other immune cells and inflammatory responses and assist in tissue repair (22). Pulmonary macrophages contain two different macrophage subsets based on anatomical location: alveolar macrophages (AMs) and interstitial macrophages (IMs). AMs are the most abundant population in pulmonary macrophages, which exist in the alveolar cavity and are directly exposed to the air and the environment and constitute the first line of defense against airborne particles and microbes (22, 23). AMs include tissue-resident alveolar macrophages (TR-AMs) and monocyte-derived alveolar macrophages (Mo-AMs). TR-AMs are derived from yolk sac-derived erythromyeloid progenitors and fetal liver monocytes, which can proliferate at a steady state to maintain self-renewal, and GM-CSF and TGF-β play an important role in this process (24–27). However, more macrophages are needed in acute inflammatory responses. When infection or injury occurs, monocytes are rapidly recruited into the alveolar cavity and develop into Mo-AMs to promote the inflammatory response and eliminate pathogens (28–30). Mo-AMs exhibit strong accumulation during early inflammation, followed by a gradual decline in their numbers, which undergo Fas-mediated cell death and local phagocytic clearance (28). A small fraction of Mo-AMs persist after infection and become phenotypically and functionally similar to TR-AMs. However, TR-AMs still survive and persist during the resolution of inflammation (28). IMs are located in the parenchyma between the microvascular endothelium and alveolar epithelium and comprise 30%-40% of lung macrophages (22, 31). IMs are initially derived from yolk sac macrophages and fetal liver monocytes and then replenished by circulating progenitor cells for their maintenance in adults (22, 32). IMs are involved in tissue remodeling and maintenance of lung homeostasis and antigen presentation as well as affect dendritic cell function to prevent airway allergy (33–35).

## 3 Polarization phenotype and polarization regulation

### 3.1 Polarization phenotype and plasticity

**Figure 1:**
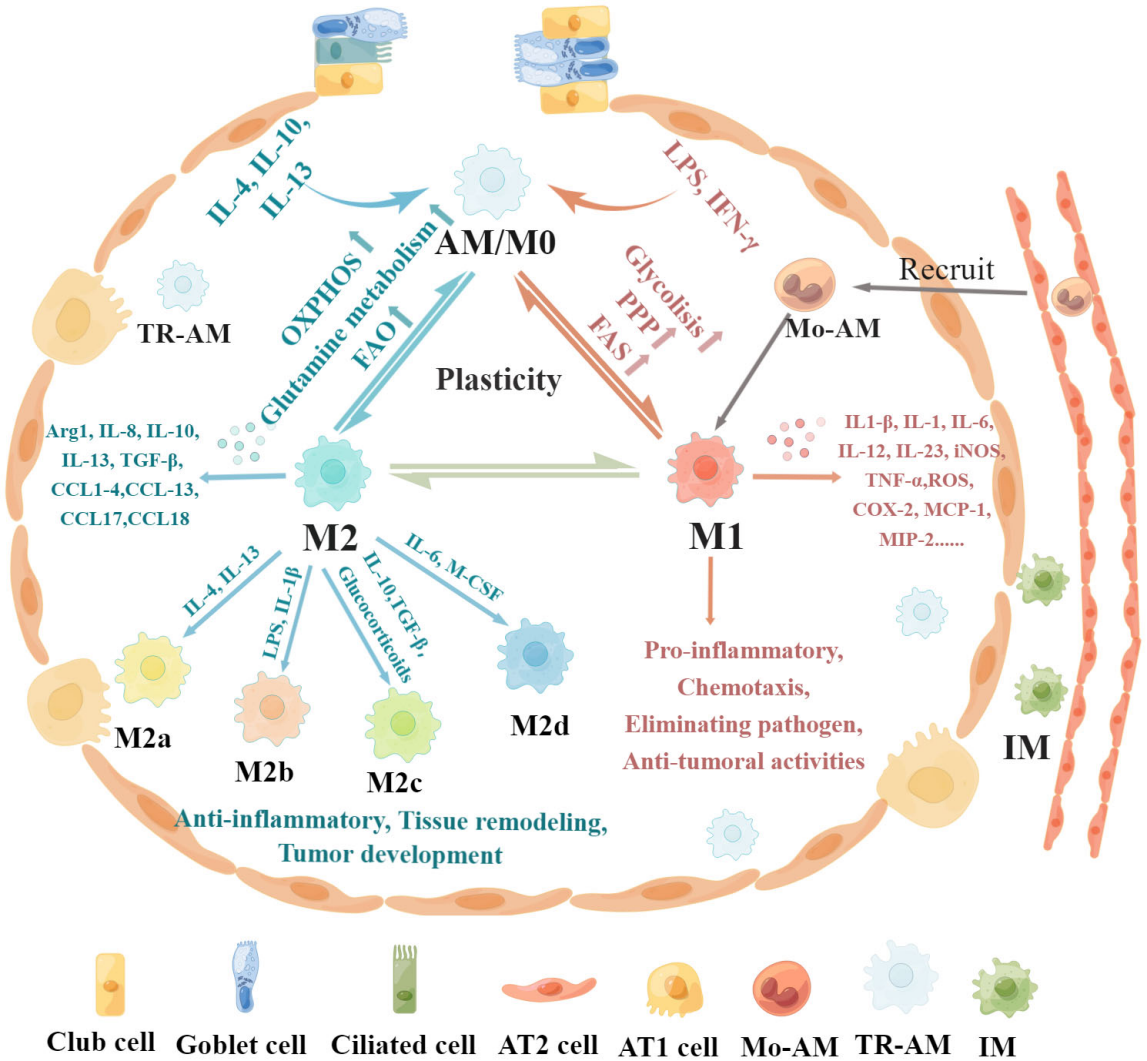
Regulatory mechanisms and functional characteristics of macrophage polarization. Non-polarized M0 macrophages can be polarized into M1 macrophages stimulated by LPS, IFN-γ, and GM-CSF, which are associated with increased glycolysis, PPP, and FAS. In addition, M0 macrophages can be polarized into M2 macrophages in the presence of IL-4, IL-13, IL-10, and M-CSF, which is related to increased OXPHOS, FAO, and glutamine metabolism. Furthermore, these polarized macrophages exhibit plasticity, as they can depolarize to M0 macrophages or exhibit the opposite phenotype through repolarization, which depends on the specific microenvironment. M1 macrophages produce pro-inflammatory cytokines such as IL-1β, iNOS, TNF-α, IL-1, IL-6, IL-12, IL-23, CCL8, CXCL1-3, CXCL-5, CXCL8-10, MCP-1, MIP-2, ROS, and COX-2, leading to pro-inflammatory responses, chemotaxis, pathogenic microorganism elimination, and antitumoral activities. M2 macrophages can be further divided into four subsets, M2a, M2b, M2c, and M2d, according to the different activating stimuli received. M2 macrophages secrete anti-inflammatory cytokines such as IL-8, IL-10, IL-13, CCL1, CCL2, CCL3, CCL4, CCL13, CCL14, CCL17, CCL18, CCL22, CCL23, CCL24, and CCL26 to exert anti-inflammatory effects, promote tissue remodeling, facilitate tumor development, and remove parasites.

Macrophage polarization refers to the activation of macrophages under the stimulation of a variety of factors and their differentiation into different phenotypes according to the state and changes in the microenvironment (14, 36). AMs have two main macrophage phenotypes: classically activated or inflammatory (M1) macrophages and alternatively activated or anti-inflammatory (M2) macrophages (37, 38). The regulatory mechanisms and functional characteristics of M1/M2 macrophages are shown in **Figure 1**. M1 macrophages constitute the first line of defense against intracellular pathogens (36). Currently, it is believed that M1 macrophages are mainly induced by lipopolysaccharide (LPS), interferon-γ (IFN-γ), and granulocyte–macrophage colony-stimulating factor (GM-CSF) (38, 39). M1 macrophages can guide acute inflammatory responses and produce a large number of pro-inflammatory cytokines such as IL-1β, inducible nitric oxide synthase (iNOS), tumor necrosis factor-α (TNF-α), IL-1, IL-6, IL-12, CCL8, IL-23, CXCL1-3, CXCL-5, CXCL8-10, monocyte chemotactic protein-1 (MCP-1), macrophage inflammatory protein 2 (MIP-2), reactive oxygen species (ROS), and cyclooxygenase 2 (COX-2) (16, 38, 40–42). M1 macrophages mainly induce Th1 response activation, exert antigen-presenting functions, and engage in pro-inflammatory responses, chemotaxis, radical formation, elimination of pathogenic microorganisms, and antitumoral activities (37, 43).

Conversely, M2 macrophages are induced in response to Th2 cytokines such as macrophage colony-stimulating factor (M-CSF), IL-4, IL-13, and IL-10 (43–45). M2 macrophages mainly express CD64 and CD209 and produce anti-inflammatory cytokines such as IL-8, IL-10, IL-13, CCL1, CCL2, CCL3, CCL4, CCL13, CCL14, CCL17, CCL18, CCL22, CCL23, CCL24, and CCL26 to exert anti-inflammatory effects, promote tissue remodeling, facilitate tumor development, and remove parasites (14, 43, 46–48). M2 macrophages can be further divided into four subsets, M2a, M2b, M2c, and M2d, according to the different activating stimuli received (41, 49). The M2a subset of macrophages can be stimulated by IL-4 or IL-13 to produce IL-10, CCL13, CCL17, and CCL22 (36, 49). These chemokines are associated with Th2 cell activation and can promote eosinophil recruitment to the lungs (50, 51). The M2b subset can be stimulated by LPS or IL-1β and produce pro-inflammatory cytokines (36, 49). The M2c subset is induced by IL-10, TGF-β, and glucocorticoids and releases high amounts of IL-10, CCL18, and CCL16 to exhibit anti-inflammatory activities (49, 52, 53). The M2d subset is induced by IL-6 and M-CSF and secretes high IL-10 and low IL-12 and TGF-β cytokine production and CXCL10, CXCL16, and CCL5 chemokines to promote angiogenesis and tumor metastasis (54, 55).

These polarized macrophages exhibit plasticity, as they can depolarize to M0 macrophages or exhibit the opposite phenotype through repolarization, which depends on the specific microenvironment (4, 43). For instance, a high αKG/succinate ratio further promotes the anti-inflammatory phenotype in M2 macrophages, whereas a low ratio enhances the pro-inflammatory phenotype in M1 macrophages (56). Specific microRNAs induced by different microenvironmental signals can regulate different patterns of macrophage polarization states by regulating transcriptional output. For instance, miR-155 promotes M1 polarization by directly inhibiting expression of BCL6, and overexpression of miR-155 can reprogram M2 macrophages into M1 macrophages (57, 58). The plasticity of epigenetic modification is an essential factor in macrophage identity and heterogeneity, which is remodeled in response to acute and polarizing stimulation (59). Histone deacetylases (HDACs) are strongly involved in M1 activation and play a prominent role in inflammatory responses (60). HDAC6 and HDAC7 are involved in the expression of pro-inflammatory genes in macrophages stimulated by LPS, and inhibition of their activity significantly limits M1 activation and the production of pro-inflammatory cytokines (61, 62). Overexpression of DNA methyltransferase 3B (DNMT3B) or loss of HDAC3 renders macrophages hyperresponsive to IL-4, skewing differentiation toward the M2 phenotype (59).

However, not all macrophages fit the classical paradigm of M1 and M2 macrophages. Tumor-associated macrophages (TAMs), one of the main types of tumor-infiltrating immune cells, are generally characterized as M2 macrophages, which are found to promote angiogenesis and invasion and tumor progression (63, 64). TCR+ macrophages, expressing the CD3/T-cell receptor (TCR)αβ complex, exist in tuberculous granulomas, atherosclerosis, and several types of cancer, which enhance phagocytosis and secrete IFN-γ, TNF, MIP-1β, and CCL2 to exhibit a specific pro-inflammatory profile (65–68).

### 3.2 Polarization regulation

**Figure 2:**
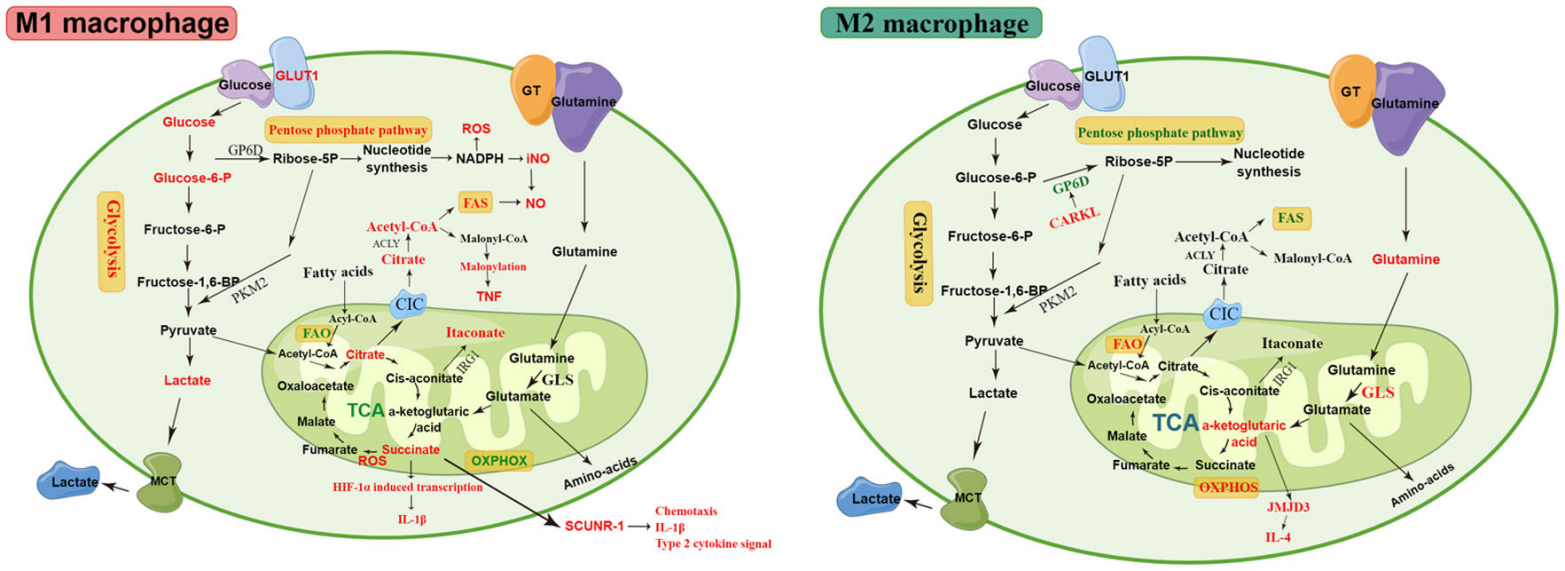
The metabolic pathways of M1 and M2 macrophages. M1 macrophages are associated with increased glycolysis, PPP, and FAS. The disturbed TCA cycle results in the accumulation of citrate and succinate. The accumulated citrate in the mitochondria of M1 macrophages is exported into the cytoplasm *via* the CIC and converted into acetyl-coenzyme A by ACLY. The downstream metabolic intermediate, acetyl-CoA, is necessary for TNF-α or IFN-γ to induce pro-inflammatory cytokines, such as NO and prostaglandin production. The malonylation response relies on citrate-derived metabolite malonyl-CoA production, which is an essential pro-inflammatory signal that promotes TNF translation and secretion by regulating glyceraldehyde 3-phosphate dehydrogenase (GAPDH). Succinate is a critical regulator of the pro-inflammatory response and regulates the expression of IL-1β via the the stabilization of hypoxia-inducible factor 1-alpha (HIF-1α)., thereby promoting LPS-induced expression of IL-1β. SDH mediates succinate oxidation and reverses electron transport, which together drive mitochondrial ROS production and induce a pro-inflammatory gene expression profile. Extracellular succinate binds to SUCNR1 and increases IL-1β production, which in turn increases SUCNR1 levels, fueling this cycle of cytokine production and perpetuating inflammation. M2 macrophages rely more heavily on OXPHOS, glutamine metabolism, and FAO than M1 macrophages. The enhancement of OXPHOS in M2 macrophages results in the continued production of ATP, leading to the upregulation of genes associated with tissue repair. The downregulated enzyme carbohydrate kinase-like protein (CARKL) causes glycolysis to feed the PPP, thereby generating nucleotides, amino acids, and NADPH, leading to an increase in ROS. αKG produced by glutamine hydrolysis promoted M2 activation through Jmjd3-dependent metabolic and epigenetic reprogramming, and a high αKG/succinate ratio further promoted the anti-inflammatory phenotype in M2 macrophages, whereas a low ratio enhances the pro-inflammatory phenotype in M1 macrophages. Elevated metabolites and processes are highlighted in red, and suppressed processes are highlighted in green.

Macrophage polarization is a complex process modulated by multiple factors, such as microRNAs (miR), proteins, glucocorticoids, and immunometabolism, involving numerous signaling pathways and transcriptional and post-transcriptional regulatory networks (14, 15, 42, 69). The precise regulatory mechanisms of macrophage polarization are still not completely understood and require further investigation. The phenotypic and functional changes in macrophages are accompanied by dramatic shifts in cell metabolism, with unique metabolic signatures related to their functional state, which is termed metabolic reprogramming (70–72). Recent studies have shown that specific metabolic pathways in macrophages are closely related to their phenotype and function, including glycolysis, tricarboxylic acid (TCA) cycle, PPP, arginine metabolism, glutamine metabolism, and fatty acid metabolism. The metabolic pathways of macrophages are shown in **Figure 2**. Some metabolic intermediates can regulate macrophage activation and effector function through various mechanisms (72).

## 4 Immunometabolism in macrophage polarization

### 4.1 Glycolysis

Glycolysis is a metabolic pathway that converts glucose to pyruvate, which plays a key role in energy metabolism, especially in the production of ATP in cells under hypoxia and other conditions. Tumor cells preferentially utilize glycolysis to produce lactic acid under normoxic conditions, known as “aerobic glycolysis.” Subsequently, several studies have observed that aerobic glycolysis is increased in pro-inflammatory macrophages (73). Although glycolysis produces far less ATP than mitochondrial OXPHOS (2 ATP *vs*. 36 ATP), glycolysis can be activated faster to match the immune response of macrophages (74–76). Multiple studies have shown that classically activated M1 macrophages in mice and humans are highly dependent on glycolysis. Van den Bossche et al. used extracellular flux analysis to demonstrate that M1 macrophages display enhanced glycolytic metabolism and reduced mitochondrial OXPHOS; conversely, M2 macrophages display enhanced mitochondrial OXPHOS (77). Rodríguez-Prados et al. showed that activation of murine peritoneal macrophages *via* the Toll-like receptor (TLR) pathway results in a hyperglycolytic phenotype, and classic activation favors the upregulation of gene expression from the glycolytic pathway and the repression of genes encoding proteins that participate in OXPHOS (73). IL-10, an anti-inflammatory cytokine that induces M2 macrophages, inhibits LPS-induced glucose uptake and glycolysis and promotes OXPHOS to oppose the switch to the metabolic program induced by inflammatory stimuli in macrophages (78).

After LPS stimulation of macrophages, transcription of the glucose transporter (GLUT1) is induced, leading to increased glucose uptake and aerobic glycolysis (79, 80). LPS specifically induces dimeric pyruvate kinase M2 (PKM2) protein expression and phosphorylation, and dimeric PKM2 interacts with HIF-1α, which is a transcription factor that contributes to both glycolysis and the induction of inflammatory genes and is critical for macrophage activation (81). HIF-1α can directly bind to the IL-1β promoter, an event that is inhibited by the activation of tetrameric PKM2 (82, 83). Activation of tetrameric PKM2 inhibits LPS-induced IL-1β production while promoting IL-10 production, thereby attenuating LPS-induced pro-inflammatory M1 macrophages and promoting the typical characteristics of M2 macrophages (82). Thus, PKM2 is a key determinant of LPS-activated macrophages that promote inflammatory responses (82). Another study demonstrated that PKM2-mediated glycolysis promotes NLRP3 and AIM2 inflammasome activation by modulating EIF2AK2 phosphorylation in LPS-primed mouse bone marrow-derived macrophages (BMDMs), consequently promoting the release of IL-1β and IL-18 and high-mobility group box 1 (HMGB1) (84). PFKFB3 is another LPS-regulated target involved in glycolytic conversion (73). PFKFB3 encodes u-PFK2, a subtype of 6-phosphofructo-2-kinase/fructose-2,6-bisphosphatase (PFK-2), which increases flux through the glycolytic pathway. PFKFB3 is a target gene of HIF-1α in response to hypoxia in human glioblastoma cell lines and mouse embryonic fibroblasts, thereby providing another mechanism by which HIF-1α promotes glycolysis (85).

Previous studies have suggested that M2 macrophages do not exhibit increased glycolysis and that aerobic glycolysis is predominantly associated with M1 macrophages. However, recent studies have shown that M2 macrophages also display an upregulated rate of glycolysis in addition to augmented mitochondrial metabolism (71). The glycolysis inhibitor 2-deoxyglucose (2-DG) (1 mM) attenuated enhanced mitochondrial respiration, significantly reduced 13C-labeled Krebs cycle metabolite levels and intracellular ATP levels, impaired IL-4-stimulated activation of early BMDMs, and reduced the expression of M2 phenotypic markers, such as Arg1, YM-1, FIZZ-1, and MRC1 (86–88). M-CSF associated with M2 polarization was found to induce similar glucose transporter expression, oxidative metabolism, mitochondrial biogenesis, and increased expression of glycolytic enzymes in macrophages compared with GM-CSF associated with the M1 phenotype (89). Glycolysis may play a more important role in M2 macrophages than previously thought, and further studies are needed.

### 4.2 Pentose phosphate pathway

PPP is an essential step in glucose metabolism, which is required for maintaining the cellular redox state and carbon homeostasis and provides precursors for nucleotide and amino acid biosynthesis. PPP can be divided into an initial oxidative phase that converts glucose 6-phosphate into carbon dioxide, ribulose 5-phosphate, and NADPH and a later non-oxidative phase that produces ribose 5-phosphate for nucleic acid synthesis and phosphate sugar precursors for amino acid synthesis (75, 90).

PPP is a major source of NADPH, which is a cofactor for NADPH-oxidase (Nox2)-dependent ROS production and is required for the generation of the antioxidant glutathione (75). The regulation of ROS levels was mediated in M1 macrophages by Cybb-encoded Nox2, which was transcriptionally upregulated in M1 macrophages and downregulated in M2 cells. Moreover, both the total pool of pentose-5-phosphates and their labeled fraction increased significantly in M1 macrophages (91). CARKL, a sedoheptulose kinase, also known as sedoheptulokinase (SHPK), catalyzes sedoheptulose to sedoheptulose-7-phosphate (S7P), which is a PPP intermediate and PPP flux restraint (92). The downregulated enzyme CARKL causes glycolysis to feed the PPP, thereby generating nucleotides, amino acids, and NADPH, leading to an increase in ROS. The CARKL expression level was rapidly downregulated in mice and humans *in vitro* and *in vivo* upon stimulation by LPS; conversely, it was upregulated in response to IL-4, leading to PPP inhibition in M2 macrophages (80). PPP was upregulated in M1 macrophages but downregulated in M2 macrophages.

### 4.3 Krebs cycle (tricarboxylic acid cycle)

The Krebs cycle, also known as the TCA cycle, is the final common pathway for the oxidation of carbohydrates, fatty acids, and amino acids and is also a source of precursors for many other biological molecules, such as non-essential amino acids, nucleotide bases, and porphyrin (93, 94). Therefore, the Krebs cycle is an important hub for cellular anabolism (gluconeogenesis and lipid synthesis) and catabolism (glycolysis).

In recent years, several studies have shown that the TCA cycle of M1 macrophages is disrupted at various points, and OXPHOS is inhibited, leading to the accumulation of citrate, itaconate, and succinate (91, 95, 96). Conversely, M2 macrophages maintain robust oxidative Krebs cycle activity, while increasing OXPHOS and ATP levels (88, 91).

#### 4.3.1 Citrate

Previous studies have demonstrated that the disruption of the TCA cycle and accumulation of citrate in M1 macrophages may be related to the transcriptional downregulation of isocitrate dehydrogenase (IDH), which catalyzes the conversion of isocitrate to α-ketoglutarate (α-KG) (91). However, Palmieri et al. showed that NO-mediated suppression of mitochondrial aconitase (ACO2), rather than IDH1, might be the TCA breakpoint in M1 macrophages, which is responsible for an increase in citrate levels and a reduction in α-KG levels (97). The mRNA and protein levels of the mitochondrial citrate carrier (CIC/SLC25a1) are markedly increased in LPS-activated macrophages (98). Inhibition of SLC25a1 in activated macrophages by genetic silencing leads to a significant reduction in ROS, NO, and prostaglandin, suggesting that the effluence of citrate from the mitochondria is an essential pro-inflammatory signal in M1 macrophage activation (98). The accumulated citrate in the mitochondria of M1 macrophages is exported into the cytoplasm *via* the CIC and converted into acetyl-coenzyme A by ATP citrate lyase (ACLY) (98). The downstream metabolic intermediate, acetyl-CoA, is necessary for TNF-α or IFN-γ to induce pro-inflammatory cytokines, such as nitric oxide (NO) and prostaglandin production (99). Inhibition of either CIC or ACLY markedly reduced prostaglandin E2 (PGE2), NO, and ROS levels (98–100). The mRNA expression of the anti-inflammatory cytokine IL-10 and IL-1 receptor antagonists in LPS-stimulated macrophages increased after ACLY inhibition (101). Another study found that the IL-4 signaling pathway cooperates with the Akt–mTORC1 pathway to regulate ACLY, resulting in increased histone acetylation and M2 gene induction (102). Furthermore, the malonylation response relies on citrate-derived metabolite malonyl-CoA production, which is an essential pro-inflammatory signal that promotes TNF translation and secretion by regulating glyceraldehyde 3-phosphate dehydrogenase (GAPDH) in response to inflammation induced by LPS (103). Furthermore, extracellular citrate may serve as a damage-associated molecular pattern (DAMP) and aggravate LPS-induced ALI by overactivating the NACHT, LRR, and PYD domain-containing protein 3 (NLRP3) inflammasome (104). Citrate and its metabolic intermediates play an important role in the M1 macrophage response, and their role in M2 macrophages needs to be further explored.

#### 4.3.2 Itaconate

Aconitase 2 catalyzes citrate to form cis-aconitate, which is decarboxylated by cis-aconitate decarboxylase, also known as immunoresponsive gene 1 (IRG1), leading to itaconate production (105, 106). Pro-inflammatory conditions can induce IRG1 expression and itaconic acid synthesis. The overexpression of IRG1 significantly inhibits LPS-induced production of TNF-α, IL-6, and IFN-β in mouse macrophages (107). In contrast, IRG1 knockout aggravates the inflammatory response in LPS-stimulated murine BMDMs and myeloid cells infected with *Mycobacterium tuberculosis* (108, 109). Itaconate has recently emerged as a regulator of macrophage functions. Itaconate reduced the production of pro-inflammatory mediators in LPS-treated macrophages, and the mechanism of this anti-inflammatory effect may be related to the inhibition of succinate dehydrogenase, blocking of IκBζ translation, and activation of Nrf2. Lampropoulou et al. reported that inhibition of itaconate-mediated succinate dehydrogenase (SDH) activity blocks mitochondrial ROS generation, inhibits NLRP3 inflammasome activation, and reduces pro-inflammatory mediator release from mouse BMDMs (108). Mills et al. demonstrated that itaconate is required for LPS-induced activation of the anti-inflammatory transcription factor Nrf2 in mouse and human macrophages (110). Itaconate directly modifies the protein KEAP1 through alkylation of cysteine residues, enabling Nrf2 to increase the expression of downstream genes with antioxidant and anti-inflammatory capacities, thereby limiting inflammatory responses and regulating type I interferons (110). Bambouskova et al. showed that itaconate and its membrane-permeable derivative dimethyl itaconate induce electrophilic stress, react with glutathione, and subsequently induce both Nrf2-dependent and Nrf2-independent responses, selectively regulating secondary transcriptional responses to TLR stimulation *via* inhibition of IκBζ protein induction (111). However, a recent study demonstrated that itaconate and itaconate derivatives (4-octyl itaconate) target JAK1 to suppress M2 macrophage polarization (112).

#### 4.3.3 Succinate

Succinate is a pro-inflammatory metabolite that accumulates during macrophage activation. Activation of macrophages using LPS leads to the accumulation of intracellular succinate through glutamine-dependent proliferation and γ-aminobutyric acid (GABA) shunt pathways (95). Increased succinate is a critical regulator of the pro-inflammatory response to LPS, both through the generation of ROS following oxidation by the electron transport chain (ETC) and *via* the stabilization of HIF-1α, a key transcription factor in the expression of pro-inflammatory genes, which in turn specifically regulates the expression of IL-1β and other HIF-1α-dependent genes and causes protein succinylation, such as malate dehydrogenase, GAPDH, glutamate carrier 1, and lactate dehydrogenase (95). Increased mitochondrial oxidation of succinate through SDH and elevation of mitochondrial membrane potential combine to drive mitochondrial ROS production and induce a pro-inflammatory gene expression profile (113). Inhibition of SDH causes succinate to accumulate and prevents the induction of a range of pro-inflammatory factors typified by IL-1β while enhancing a range of anti-inflammatory factors typified by IL-1RA and IL-10 (113). Thus, SDH enhances the oxidation of succinate, is required for the induction of pro-inflammatory genes, and simultaneously limits the induction of anti-inflammatory genes. The succinate receptor SUCNR1/GPR91 is a G protein-coupled cell surface sensor for extracellular succinate. Littlewood-Evans et al. proposed a mechanism for SUCNR1-driven autocrine and paracrine enhancement of IL-1β release from activated macrophages (114, 115). Endogenous TLR ligands activate macrophages locally, resulting in enhanced glycolysis and increased intracellular succinate levels. Simultaneously, succinate is released into the extracellular milieu, where it binds to SUCNR1 and increases IL-1β production from the same or adjacent SUCNR1-expressing cells, which in turn increases SUCNR1 levels, fueling this cycle of cytokine production and perpetuating inflammation (115). Gut microbiota-derived succinate exacerbates intestinal ischemia/reperfusion-induced ALI through SUCNR1-dependent M1 polarization, and plasma succinate levels are significantly correlated with ALI (116). Succinate and its receptors play an important role in macrophage metabolism and may serve as important targets for inflammatory diseases in the future.

### 4.4 Glutamine metabolism

Glutamine, serving as a carbon and nitrogen source for metabolic reprogramming of M1 macrophages, can be broken down to produce glutamate and α-KG, the latter of which enters the TCA cycle to provide energy. Furthermore, glutamine metabolism represents an important metabolic module governing the alternative activation of macrophages in response to IL-4 (91). Palmieri et al. reported that inhibition of glutamine synthetase skewed M2-polarized macrophages toward the M1-like phenotype, characterized by decreased intracellular glutamine and increased succinate with enhanced glucose flux through glycolysis, showing an enhanced capacity to induce T-cell recruitment and reduced T-cell suppressive potential, which could be partly related to the activation of HIF-1α (117). Production of αKG by glutamine hydrolysis is important for the alternative activation of macrophages (56). αKG promotes M2 activation through Jmjd3-dependent metabolic and epigenetic reprogramming, and a high αKG/succinate ratio further promotes the anti-inflammatory phenotype in M2 macrophages, whereas a low ratio enhances the pro-inflammatory phenotype in M1 macrophages (56). Moreover, αKG inhibits the nuclear factor-κB (NF-κB) pathway through prolyl hydroxylase (PHD)-dependent proline hydroxylation of protein kinase IKKβ, thereby impairing the pro-inflammatory response of M1 macrophages (56). The peroxisome proliferator-activated receptor γ (PPARγ) is involved in the alternative activation of macrophages (56, 118, 119). PPARγ is required for IL-4-induced M2 macrophage respiration, and the absence of PPARγ dramatically affects glutamine oxidation (120). Unstimulated macrophages lacking PPARγ contain elevated levels of the inflammation-associated metabolite itaconate and express a pro-inflammatory transcriptome (120).

### 4.5 Arginine metabolism

Arginine metabolism is a complex process characterized differently in M1 and M2 macrophages. M1 macrophages express NO synthase, which metabolizes arginine to NO and citrulline, and citrulline can be reused for efficient NO synthesis *via* the citrulline–NO cycle (121). M2 macrophages are characterized by the expression of arginase, which hydrolyzes arginine to generate ornithine and urea, a precursor of l-proline and polyamines involved in tissue repair and wound healing (122). There are two isozymes of arginase: arginase I and arginase II. The hepatic urea cycle arginase I is expressed as a cytosolic enzyme, while human granulocyte arginase I is found in the granular compartment and arginase II is found as a mitochondrial enzyme (123). Arg1 inhibits NO-mediated inflammatory pathways by competing with iNOS for l-arginine. A recent study demonstrated that renal tubular cells apically exposed to dead cell debris induce reparative macrophage activation, expressing Arg1, which is required for the S3 tubular cell proliferative response that promotes renal repair after ischemia–reperfusion injury (124). Moreover, Zhang et al. found that polarization of M2a macrophages promotes the expression of Arg1, which restores axonal regeneration and promotes the structural and functional recovery of the contused spinal cord (125). Hardbower et al. reported that Arg2 deletion leads to enhanced M1 macrophage activation, pro-inflammatory cytokine expression, and immune cell-derived chemokine production (126). Arg2 is essential for the IL-10-mediated increase in mitochondrial oxidative metabolism and succinate dehydrogenase/complex II activity and the decrease in the inflammatory mediators succinate, HIF-1α, and IL-1β (127). Therefore, arginine metabolism plays an important role in the anti-inflammatory effects and tissue repair of macrophages.

### 4.6 Fatty acid metabolism

The TCA cycle of M1 macrophages is disrupted, causing the accumulation of citrate, which is exported from the mitochondria to the cytoplasm to generate acetyl-CoA *via* ACLY, which fuels the *de-novo* synthesis of cholesterol and FA (128). These steps involve ACLY, acetyl-CoA carboxylase (ACC), fatty acid synthase (FASN), desaturases, and elongation proteins. Fatty acids tightly couple glucose and lipid metabolism *via* the *de-novo* FA synthesis pathway, supporting cell adaptation to environmental changes. The FAO pathway produces acetyl-CoA, NADH, and FADH2, which are further used in the TCA cycle and ETC to generate large amounts of ATP, which is thought to promote M2 polarization. This process is coordinated by multiple enzymes such as fatty acyl CoA synthetase, carnitine palmitoyltransferase I (CPT1), and carnitine palmitoyltransferase II (CPT2).

#### 4.6.1 Fatty acid synthesis

Fatty acid synthesis is closely related to the pro-inflammatory function of macrophages. Several studies have suggested that cellular fatty acid synthesis (e.g., synthesis of triglycerides and cholesteryl esters) is activated during inflammation (129–131). Saturated fatty acids (SFAs) can induce inflammation either extracellularly by activating TLR signaling or intracellularly *via* products of SFA metabolism, thereby inducing NF-κB activation and the expression of COX-2 and other inflammatory markers (132). Moon et al. demonstrated that mitochondrial uncoupling protein-2 regulates the NLRP3 inflammasome by inducing the lipid synthesis pathway during macrophage activation (133). The NLRP3 inflammasome finely regulates the activation of caspase-1 and the production and secretion of potent pro-inflammatory cytokines such as IL-1β and IL-18 (134). The fatty acid metabolism-immunity nexus (FAMIN; LACC1, C13orf31) forms a complex with FASN on peroxisomes to concomitantly drive high levels of FAO and glycolysis, controlled inflammasome activation, mitochondrial and NADPH-oxidase-dependent production of ROS, TLR-dependent signaling, cytokine secretion, and the bactericidal activity of macrophages (135). Impaired FAMIN compromises both classically activated M1 macrophages and alternatively activated M2 macrophages (135–137).

#### 4.6.2 FAO

LPS-treated macrophages stimulated fatty acid uptake into the cell, which was accompanied by a marked increase in the expression of CD36, a protein that transports fatty acids (131). The ability of LPS-treated macrophages to oxidize fatty acids to CO_2_ was greatly reduced, which was associated with reduced expression of CPT1α and CPT1β proteins that promote fatty acid entry into the mitochondria for oxidation (131). Lv et al. demonstrated that didymin, a flavonoid constituent, strengthened FAO rather than glycolysis by inducing Hadhb expression, resulting in the conversion of M1-like macrophages toward M2-like macrophages and eventually alleviating colitis (138). Hohensinner et al. showed that the pharmacological inhibition of FAO in macrophages reduced NLRP3 activation, leading to reduced levels of the pro-inflammatory cytokine IL-1β in macrophages, thereby suppressing the inflammatory response (139). Namgaladze et al. showed that IL-4-induced M2 polarization of murine macrophages in response to IL-4 is associated with the increase in mitochondrial oxidative metabolism and FAO (140). However, in human macrophages, IL-4 causes only moderate changes in mitochondrial oxidative metabolism and FAO; attenuating FAO had no effect on IL-4-induced polarization-associated gene expression (140). FAO is not essential for M2 activation, and further research is needed to clarify the underlying mechanism.

## 5 Macrophage polarization and ALI/ARDS

Macrophages participate in the pathogenesis of ALI/ARDS, including the regulation of inflammatory responses and the repair of damaged lung tissues (12, 13). M1 macrophages are involved in the acute inflammatory response and exudative phase of ALI/ARDS by secreting various pro-inflammatory cytokines and recruiting neutrophils from the circulation into the lungs and alveoli, leading to the progression of inflammation and enhanced lung injury (4, 16). However, M2 macrophages are mainly associated with the resolution of inflammation and the recovery phase in ALI/ARDS by producing anti-inflammatory cytokines and limiting the levels of pro-inflammatory cytokines to alleviate the inflammation response and promote lung tissue repair (4, 16). Excessive M2 polarization may result in a pathological fibroproliferative response and pulmonary fibrosis (4). Macrophage polarization can be modulated by multiple factors, and immunometabolism and its metabolic intermediates are one of the most important factors. Altering the direction of macrophage polarization and limiting excessive pro-inflammatory responses by modulating immunometabolism and its metabolic intermediates may greatly affect the prognosis of ALI/ARDS.

Recent studies have shown that several compounds affect macrophage polarization by modulating immunometabolism and its metabolic intermediates. N-phenethyl-5-phenylpicolinamide (N5P) is a newly synthesized HIF-1α inhibitor. Du et al. showed that N5P effectively reduced HIF-1α, GLUT1, HK2, ASIC1a, IL-1β, and IL-6 expression levels in LPS-induced ALI, which may alleviate inflammation in ALI through the HIF-1α/glycolysis/ASIC1a signaling pathway (141). Zhong et al. demonstrated that inhibition of glycolysis by 2-DG pronouncedly attenuated lung tissue pathological injury, oxidative stress, and the expression of pro-inflammatory factors in ALI mice induced by LPS (142). Tanshinone IIA (Tan-IIA), a major constituent of *Salvia miltiorrhiza* Bunge, significantly decreased succinate-boosted IL-1β and IL-6 production, accompanied by upregulation of IL-1RA and IL-10 release *via* inhibition of SDH and reduced mitochondrial ROS production, leading to remarkably attenuated LPS-induced acute inflammatory responses (143). Dimethyl malonate (DMM), which is rapidly hydrolyzed in cells to form malonate, is a competitive inhibitor of succinate oxidation by SDH (113). Mills et al. found that inhibition of SDH with DMM was effective in an LPS-induced sepsis mouse model, where it decreased the serum levels of IL-1β and boosted IL-10, but had no significant effect on TNF-α (113). In a mouse model of LPS-induced peritonitis, Lauterbach et al. revealed decreased protein levels of IL-6 and IL-12 in the peritoneum and serum by inhibiting ACLY using BMS 303141, suggesting that ACLY inhibition was able to alter the local and systemic inflammatory profiles (144). Inhibition of SLC25a1 in activated macrophages with the inhibitor 1,2,3-benzentricarboxylic acid (BTA) or through genetic silencing reduced accumulated citrate output from the mitochondria to the cytoplasm *via* SLC25a1, leading to a marked reduction in ROS, NO, and prostaglandin production (98). Production of αKG by glutamine hydrolysis is important for the alternative activation of macrophages. Liu et al. found that α-KG pretreatment diminished the lung damage score, inhibited the secretion of inflammatory cytokines in sera, suppressed M1 marker gene expression (IL-1β, IL-6, and TNF-α) and enhanced M2 marker gene expression (Arg1) to attenuate LPS-induced ALI/ARDS in a mouse model (145). Furthermore, in other diseases mediated by inflammatory responses, it is also promising to reduce the severity of disease by regulating the polarization of macrophages through immunometabolism. Didymin, a flavonoid constituent, strengthens FAO rather than glycolysis, resulting in the conversion of M1- toward M2-like macrophages, but does not alter the polarization of M2-like macrophages and remarkably alleviates the clinical symptoms of acute and chronic colitis in mice (138). Targeting immunometabolism to regulate macrophage polarization for ALI/ARDS treatment has been proven effective and promising in many studies. However, the current relevant studies are insufficient, and almost all of them are cell experiments or animal experiments. Further studies are needed to elucidate the role of immunometabolism and its metabolic intermediates in macrophage polarization and ALI/ARDS, which may represent new targets and therapeutic directions.

## 6 Conclusion

In the pathogenesis of ALI/ARDS, M1 macrophages engage in pro-inflammatory responses, chemotaxis, radical formation, and elimination of pathogenic microorganisms, while M2 macrophages exert anti-inflammatory effects and promote tissue remodeling and lung repair. Polarized macrophages exhibit plasticity; thus, targeting macrophage polarization has potential benefits for alleviating inflammation and promoting lung repair in ALI/ARDS. Immunometabolism is a significant and complex process involving multiple metabolic pathways and metabolic intermediates, such as glycolysis, the Krebs cycle, the pentose phosphate pathway, amino acid metabolism, and fatty acid metabolism. A growing body of literature on immunometabolism has demonstrated the essential role of immunometabolism and its metabolic intermediates in macrophage polarization. Therefore, the regulation of macrophage immunometabolism to alter macrophage polarization may be a new direction for the treatment of ALI/ARDS.

## Author contributions

Conceptualization: LW and DW Investigation: XT and HF. Resources: TZ and YM. Writing—original draft preparation: LW and DW. Writing—review and editing: XT and HF. Supervision: HF All authors contributed to the article and approved the submitted version.
